# Structural, magnetic and electronic properties of two dimensional NdN: an *ab initio* study

**DOI:** 10.1039/c9ra07429e

**Published:** 2019-11-04

**Authors:** S. Assa Aravindh, Iman S. Roqan

**Affiliations:** King Abdullah University of Science and Technology (KAUST), Division of Physical Sciences and Engineering Saudi Arabia iman.roqan@kaust.edu.sa

## Abstract

The peculiar magnetic properties of rare earth nitrides (RENs) make them suitable for a wide range of applications. Here, we report on a density functional theory (DFT) study of an interesting member of the family, two-dimensional (2D) NdN film, using the generalized gradient approximation (GGA), including the Hubbard (*U*) parameter. We consider different film thicknesses, taking into account the effects of N vacancies (*V*_N_) and dopants (C and O). Formation energy values show that, even though N vacancy is the predominant defect, C and O dopants are also probable impurities in these films. Individual Nd and N magnetic moments oscillate in the presence of *V*_N_ and dopants owing to the induced lattice distortions. The density of states calculations show that the 2D NdN film has a semi-metallic nature, while the f orbitals are separated into fully filled and empty bands. A magnetic anisotropy energy of ∼50 μeV is obtained, and the easy axis aligns along the film orientation as the film thickness increases, revealing that such films are ideal candidates for spintronic applications.

## Abstract 2

We found that the peculiar magnetic properties of rare earth nitrides (RENs), mainly NdN films, make them suitable for a wide range of applications.

## Graphical abstract

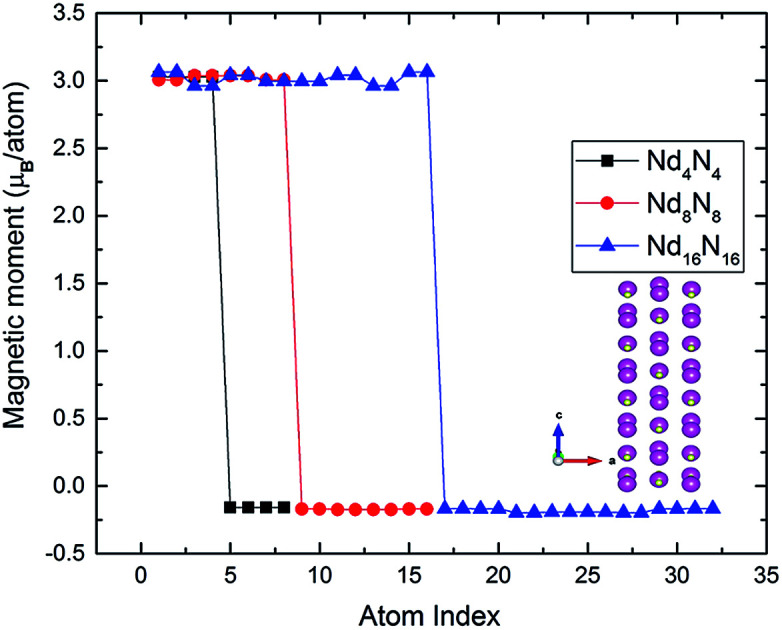

## 1. Introduction

Elements in the rare-earth nitride (REN) family are interesting from both a fundamental and a practical point of view, owing to their partially occupied 4f sub-shell, which in turn leads to peculiar magnetic and electronic properties.^1–6^ The resulting ferromagnetic (FM) and diverse conducting properties, ranging from half-metallic through insulating to semi-metallic, make them attractive candidates for spintronic devices,^5^ which contrasts with dilute magnetic semiconductors (DMS). In addition, while the magnetic and electronic properties of DMS materials are primarily governed by the presence of dopants or defects,^7–12^ RENs do not require doped foreign atoms or large hole concentrations to generate magnetic interactions.^5,13^ Moreover, RENs are the only stable elements whose f orbitals are more than marginally filled, leading to large spin and orbital moments. Taking advantage of these characteristics, RENs have been extensively studied, aiming to identify prototypical spintronic devices.^5^ For example, the possibility of obtaining a GdN-based spin filter has been reported.^14^ Apart from magnetic properties, the electronic and transport properties of RENs also present many ambiguities.^5,13^ Even for compounds having similar structures, insulating to metallic behavior was reported,^5^ which was attributed to the highly reactive nature of these materials and the ease with which nitrogen vacancies form.^5^

Among this family of materials, NdN remains the least studied, due to the difficulty in preparing bulk samples with high density and purity.^5,13,15^ The main obstacles are the ease of nitrogen vacancy (*V*_N_) formation, along with easy decomposition into oxides or hydroxides. Interestingly, NdN possesses trivalent state only, while some other members of the family such as Sm and Eu act partly or completely as divalent cations.^5^ This is reflected in the magnetic properties, as NdN is the sole ferromagnetic nitride in the Nd series, as in the REN family, the nearest neighbor exchange parameter changes with the increase in anion size, from positive for NdN to negative for other RENs.^1^ Coming to the existing studies on NdN, the cubic lattice parameters of bulk NdN have been determined experimentally.^5^ Moreover, density functional theory (DFT) calculations have shown that bulk NdN is stable in the cubic structure (B1) with FM ground state and half-metallic properties.^13,16^ Further, the half-metallic ground state of bulk NdN was predicted by Hao *et al.*, by the calculation of elastic properties.^17^ Anton *et al.*^18^ have successfully synthesized and reported semiconducting behavior and orbital dominant magnetic moment for NdN thin films. Nevertheless, paramagnetic phase was also reported in earlier studies of bulk NdN, with a band gap of 0.8 eV.^3^ Therefore, as the reported magnetic properties of this important REN material are inconsistent, further investigations are needed. In particular, the prominent magnetic properties of NdN, such as increased magnetic moments, orbital moment domination and its effect on the magnetic anisotropy energy, and the easy axis of magnetization have not been explored for the thin films.

In this paper, we address these gaps by a DFT study of the magnetic and electronic properties of two-dimensional (2D) NdN oriented along the NaCl (001) direction. The spin-orbit coupling (SOC) effects are included in calculations to estimate the spin and orbital contribution to magnetism as well as to determine axis preference of magnetization.

## II. Computational details

We use the plane-wave pseudopotential code Vienna Abinitio Simulation Package (VASP)^19,20^ to carry out the DFT calculations. The exchange and correlation functional were described using generalized gradient approximation (GGA) in the spin-polarized approach,^21,22^ while projected augmented wave (PAW) framework was employed for the pseudopotentials. Energy and force tolerances of 10^−6^ eV and 0.001 eV Å^−1^, respectively, were adopted for the relaxation of atomic coordinates. A kinetic energy cut-off of 400 eV was used to expand the plane waves included in the basis set, and the Brillouin zone was sampled using a Monkhorst–Pack *k*-grid of 12 × 12 × 2 dimensions. To account for the strong electronic correlations among rare earth elements, the Hubbard parameter with onsite Coulomb corrections was adopted, where *U* = 7.609 and *J* = 0.987.^23^ The cubic lattice parameters of bulk NdN have been determined experimentally with a band gap of 0.8 eV.^24^ The ground state of bulk NdN is FCC NaCl structure, with lattice parameter of 5.15 Å.^5^ We calculated lattice parameter value of 5.2 Å for bulk NdN, which is in reasonable agreement with the experimental results.^5^ This relaxed lattice parameter was used to model the 2D NdN films, comprising of eight atomic layers, while vacuum of 10 Å was applied along the *z*-direction to avoid interaction between the periodically repeated images.

## III. Results and discussion

**Fig. 1:**
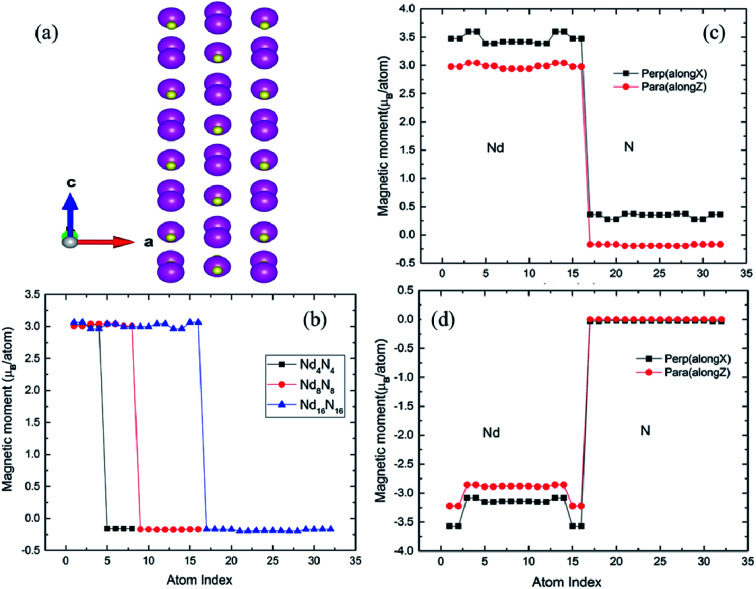
(a) Relaxed structure of 2D NdN film with eight atomic layers. The pink- and gold-colored atoms indicate Nd and N, respectively. (b) Spin magnetic moments of NdN as a function of layer thickness. (c) Spin and (d) orbital magnetic moments for the 2D Nd_16_N_16_ layers under parallel and perpendicular spin magnetic moment orientation.

The 2D NdN films were constructed along the NaCl (001) orientation and was subjected to atomic relaxation. The optimized structure shown in Fig. 1(a) reveals significant relaxation in the atomic layers. After the relaxation, the Nd–N and N–N bond lengths were 2.53 Å and 3.37 Å, respectively, whereas the corresponding bulk values were 2.51 Å and 3.54 Å. As Natali *et al.* have reported that *V*_N_ is an unavoidable defect in bulk NdN during growth,^5^ we quantify the presence of *V*_N_ and other impurities in the 2D films, by calculating the formation energy (*E*^f^), using the expression:

1 *E*^f^ = *E*(Nd_16_N_16−*X*_) − *E*(Nd_16_N_16_) ± *μ*_*X*_ + *q*(*E*_F_)

where the first two terms represent the total energy of the supercell containing the defect and that of the perfect supercell, respectively. The third term denotes the chemical potential of the atom that is added or removed, the value of which depends on the experimental growth conditions. Here, *q* and *E*_F_ denote the charge and Fermi energy, respectively. However, it should be noted that, in the present study, all calculations were conducted in the *q* = 0 state. The chemical potentials (*μ*) of the impurities were calculated from the total energy of their respective molecular states. Using eqn (1), we also calculated the *E*^f^ of C and O impurities by substituting for the N atom in the films. The inclusion of C and O dopants in the calculations was motivated by the fact that they are unavoidable impurities during preparation and their presence often alters the properties of thin films.^5^ However, Nd vacancies are not included in our calculations because they are less likely to be formed compared to the lighter N atom vacancies.

The *E*^f^ values of *V*_N_ are reported in Table 1, whereby a negative value indicates a high likelihood of occurrence of defects in the material. The *E*^f^ calculations demonstrate that both C (*E*^f^ = −1.68 eV) and O (*E*^f^ = −2.96 eV) impurities are also favorable stable defects. However, it is interesting to note that *V*_N_ forms much more easily (*E*^f^ = −5.34 eV) compared to C and O and is consequently the most predominant defect. The negative formation energies indicate that, these defects can easily form while preparation, and it might be very difficult to grow defect free NdN thin films. For decades, it has been a major obstacle, as there existed hardly any method which could produce REN films devoid of *V*_N_ and prevent decomposition in air to oxides and hydroxides.^25–27^ It is the recent advances in thin film technology such as ultra-high vacuum based methods, that made the controlled growth and high quality REN films a reality.^28^ Nevertheless, our results render a qualitative assessment of defect formation in NdN thin films. As we found that *V*_N_ is the prominent defect, other defects are not considered in the subsequent magnetic calculations.

**Table 1:** Formation energy of N vacancy, O and C dopants in the 2D Nd_16_N_16_ layers

Configuration	Formation energy (eV)
Nd_16_N_15_	−5.34
Nd_16_N_15_O	−2.96
Nd_16_N_15_C	−1.68

It is known that materials of particular interest for magnetic or spintronic applications are those associated with large spin magnetic moments. The interesting fact about bulk RENs is that they possess high magnetic moments combined with electronic structure corresponding to semiconductors.^5^ However, their half-metallic and semi-metallic nature has also been reported.^5^ In this work, the magnetic moments of films with different number of layers (Nd_4_N_4_, Nd_8_N_8_, Nd_16_N_16_) were compared, as shown in Fig. 1(b). It is evident that, for the thinnest film, the magnetic moments of both Nd and N remain unchanged. As the film thickness increases, the magnetic moments of Nd and N atoms in different layers exhibit oscillations, whereby those of the innermost layers are decreased, while those of outer layers are increased. This phenomenon can be attributed to the more bulk-like environment in the inner layers, which in turn leads to reduced magnetic moments. On the other hand, the atoms in the outer layers are subjected to surface effects, which enhances their magnetic moment due to the changes in bonding environment.

It is also noteworthy that, according to the extant studies, the magnetic moments are orbital-dominant for some of the members in the REN family.^5^ For example, SmN and HoN exhibit negative magnetization, owing to their negative orbital moment.^5^ Hence, to understand the magnetic behavior of NdN, it is significantly important to include spin-orbital coupling (SOC), which discerns the contributions of both spin and orbital magnetic moments to the total magnetization of the system. Hence, non-collinear calculations were performed by initializing the spin magnetic moments in both parallel and perpendicular directions to the film orientation. The spin and orbital moments calculated for the Nd_16_N_16_ films are shown in Fig. 1(c) and (d). The spin magnetic moments of both Nd and N increase when oriented perpendicular to the film growth direction compared to parallel orientation, as shown in Fig. 1(c). For the perpendicular orientation of spin moments, the Nd magnetic moments reach values as high as ∼3.7 *μ*_B_ per atom, whereas the value of magnetic moments of Nd in bulk NdN is 3 *μ*_B_ per atom. The corresponding value for the N atom in bulk NdN is −0.19 *μ*_B_ per atom, while in 2D NdN, N magnetic moments are positive, reaching about 0.5 *μ*_B_ per atom. The orbital moments are also presented for both parallel and perpendicular orientations in Fig. 1(d), indicating that the films present large and negative orbital moment values for the Nd atoms as expected from the REN family. The orbital moment of N atoms is very small and is almost consistent for both parallel and perpendicular orientation of spin moments.

To understand the preferential orientation of magnetic moments to a particular axis, the energy required to change the orientation from easy axis to hard axis, —denoted as magnetic anisotropy energy (MAE)—should be investigated. The origin of the magnetic anisotropy is SOC, and therefore the possibility of manipulating the orbital and spin moment of a crystal is crucial. Mainly, in low-dimensional systems, SOC plays a major role in controlling the MAE. Thus, we calculated MAE by adopting the total energy difference approach such that, MAE = *E*_para_ − *E*_perp_, whereby the spin moments were aligned parallel (*E*_para_) and perpendicular (*E*_perp_) to the orientation of the 2D NdN. From this definition, a negative value of MAE implies in-plane orientation and *vice versa*. The obtained MAE value increases with thickness of the 2D layers, and the orientation change, as shown in Table 2. For the thinnest layer, (0.25 nm thickness), a MAE value of 22 μeV per atom was obtained, indicating preferential perpendicular orientation. However, once the thickness of the film surpasses 0.75 nm, MAE becomes negative, whereby its absolute value steadily increases to ∼50 μeV per atom, signifying preference for parallel spin orientation.

**Table 2:** The magnetic anisotropy energy (MAE) for the 2D NdN layers for different thicknesses

Layer thickness (nm)	Configuration	MAE (μeV)	*m*_*L*_perp__ (*μ*_B_)	*m*_*L*_para__ (*μ*_B_)	Preferred spin orientation
0.25	Nd_4_N_4_	22.0	0.07	0.06	Perpendicular
0.75	Nd_8_N_8_	−40.0	0.05	0.07	Parallel
1.7	Nd_16_N_16_	−51.0	0.07	0.08	Parallel
1.7	Nd_16_N_15_	−55.0	0.07	0.08	Parallel

The aforementioned variation in MAE with layer thickness was analyzed in relation to the anisotropy of orbital moments. These two anisotropies are interrelated by Bruno's rule,^29–31^ according to which the difference in orbital moments along two different orientations is given as by Δ*m*_L_ = *L*_para_ − *L*_perp_. The orbital moments pertaining to layers of different thickness reported in Table 2 indicate that their values are greater along the perpendicular orientation (*L*_perp_) for the 0.25 nm film only. On the other hand, slightly increased *L*_para_ moments were observed along the parallel orientation for other thicknesses, which is congruent with the obtained MAE values. These un-quenched values of orbital moments in the layers arise due to the existence of asymmetric interactions compared to the bulk. These results reveal a unique magnetic anisotropic behavior of 2D NdN film, which is in line with the experimental results obtained for NdN-related materials (Nd_2_Fe_l7_N_*x*_).^32^ Since large MAE value is essential for spintronic applications to avoid the spin-flip transition, these results indicate that 2D NdN films are promising candidates for data storage as well as for other potential smart magnetic applications, as its MAE can be tuned by varying the number of layers.

**Fig. 2:**
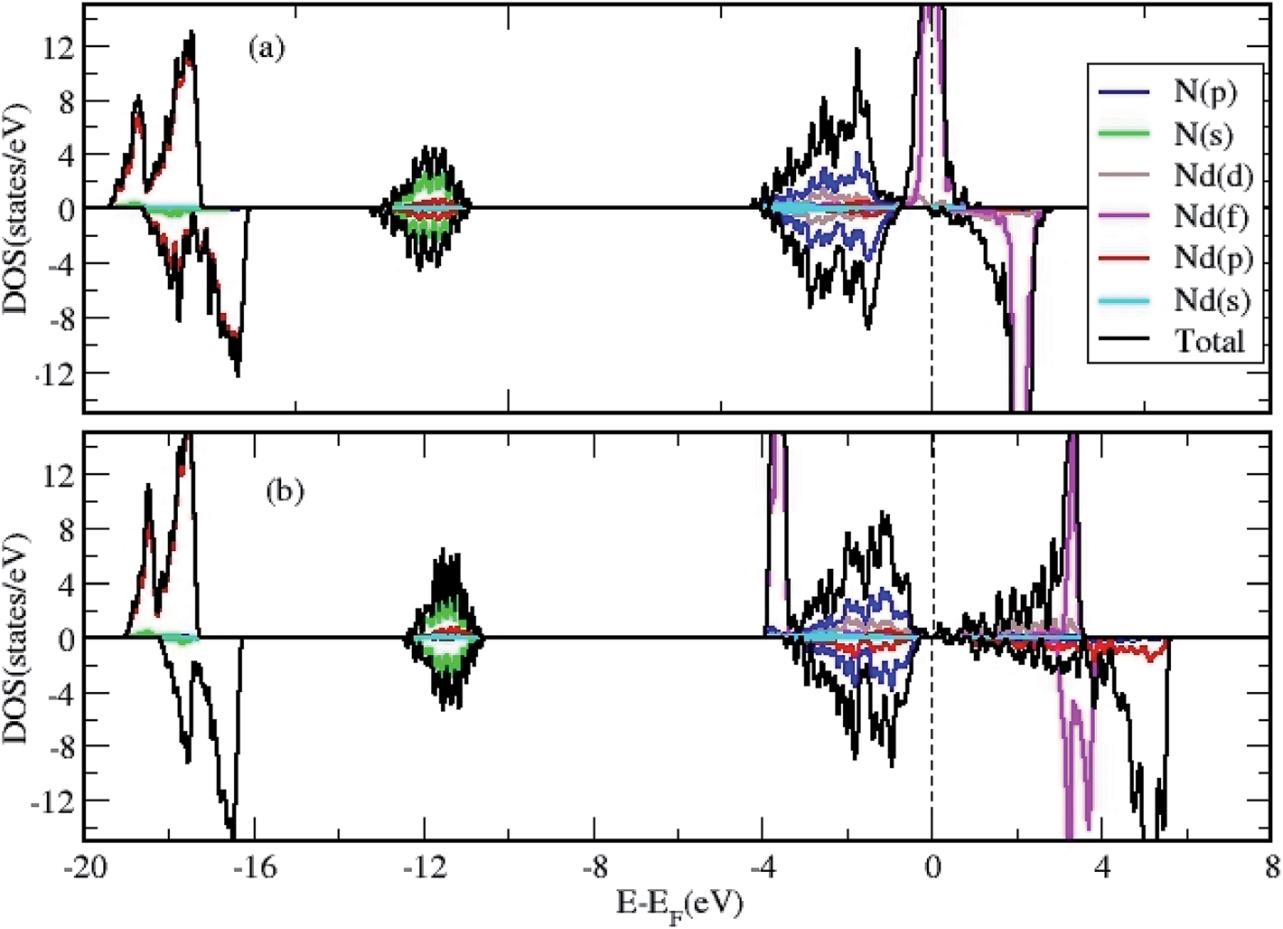
The total and projected DOS of bulk NdN calculated by (a) GGA and (b) GGA+*U* approximation.

**Fig. 3:**
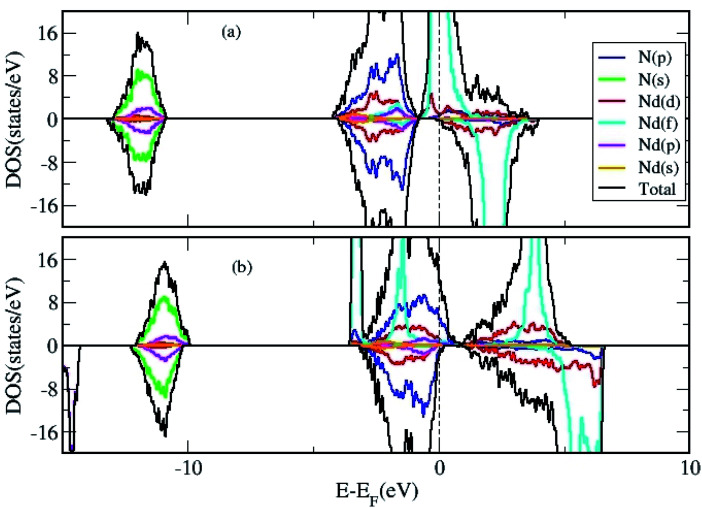
The total and projected DOS of 2D Nd_16_N_16_ layers calculated using (a) GGA and (b) GGA+*U* approximation.

We calculated the density of states (DOS) to gain further insight in to the properties of 2D NdN layers. First, we have analyzed effect of *U* parameter on the electronic structure of bulk NdN. The findings indicate that, when *U* is not considered in the calculation, bulk NdN exhibits metallic behavior, whereas semiconducting behavior with a very narrow gap is observed when the *U* parameter is incorporated, as shown in Fig. 2(a) and (b), respectively. The projected DOS (PDOS) is also presented, signifying the respective atomic contributions. This indicates that to account for the strong electronic correlations in these materials, approximations beyond standard DFT must be included to obtain meaningful results. Further, the DOS of the 2D Nd_16_N_16_ layers were calculated, and shown in Fig. 3(a) and (b), depicting the total density of states (TDOS) and PDOS obtained using GGA and GGA+*U*, respectively. It is evident that the 2D layers exhibit entirely different conducting properties under GGA and GGA+*U* approximations, emphasizing the importance of selecting the correct formalism for determining the electronic properties of RENs. When GGA is adopted, the 2D layers show half-metallic behavior similar to the bulk NdN. A half-metallic material is one, that conducts electrons of one spin orientation and acts as insulator for electrons possessing opposite spin.^33^ Hence in a typical half-metal, the valence band for one spin direction will be partially filled and presents a gap in the DOS for the other spin direction.^33^

Previously, for some RENs such as EuN, half metallicity and for others, metallic behavior is reported.^34^ However, we found that when the correction of GGA+*U* scheme is employed, the *E*_F_ shifts compared to those obtained by GGA calculations, and an overlap emerges between the valence and the conduction bands, as shown in Fig. 3(b). Under the GGA+*U* approach, a small effective DOS emerges at the *E*_F_, indicating the signature of semi-metallic behavior. A semi metal is characterized by a very small overlap between the bottom of the conduction band and the bottom of the valence band, and the RENs have been shown to exhibit semi-metallic properties.^7^ Thus, compared to the half-metallic nature obtained by using GGA approach, where one spin channel is completely insulating for electrons of the opposite spin, GGA+*U* renders entirely different electronic properties for NdN thin films. It is evident that the inclusion of parameter *U* changes the position of Nd f states, which shift away from the Fermi level (*E*_F_). Moreover, Fig. 3(b) shows that there is a small effective DOS at the *E*_F_, which arises from the contribution of the N-p states. The potential for hybridization of N-p states with d and f states of Nd is also evident from the PDOS depicted in Fig. 3(b).

**Fig. 4:**
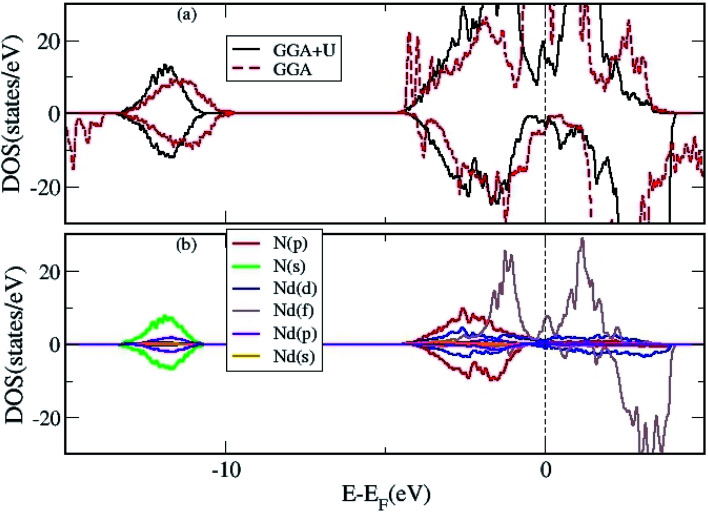
(a) The total DOS of 2D Nd_16_N_15_ layers calculated using both GGA and GGA+*U* approximation, and (b) the projected DOS using the GGA+*U* approximation.

Lastly, the DOS were calculated for 2D Nd_16_N_15_ layers in the presence of *V*_N_ to elucidate its effect on the conducting and magnetic properties, as the carriers provided by the vacancy will affect the electronic properties. The TDOS calculated using GGA and GGA+*U* are shown in Fig. 4(a), whereas the PDOS calculated using GGA+*U* are shown in Fig. 4(b). These findings indicate that the charge carriers provided by *V*_N_ can mediate magnetic interactions in the layers. The f band no longer overlaps at the *E*_F_, and the semi-metallic nature of the NdN films prevails with the inclusion of the *U* parameter. These results demonstrate the origin of the conductivity and magnetism exhibited by 2D NdN films, which have not been previously studied in details experimentally.

## IV. Conclusions

We have carried DFT studies on 2D NdN films oriented along the NaCl (001) direction. We found that *V*_N_ is a favorable defect compared to external impurities and the thin films exhibit semi-metallic nature according to the GGA+*U* approximation, while GGA predicted half-metallic character. The f orbitals are appeared to be pinned around the *E*_F_ when GGA approach was applied, whereas using GGA+*U* the f orbitals were shifted into filled and empty bands. NdN films show magnetic anisotropy of about ∼50 μeV per atom with an easy axis along the film orientation as the film thickness increases. These results can add value to the existing and ongoing studies on REN thin films, as well as benefit further efforts to exploit their properties in the context of magnetic and spintronic applications.

## Conflicts of interest

There are no conflicts of interest to declare.

## Supplementary Material
